# Association between the Use of Folic Acid Supplements during Pregnancy and Children’s Cognitive Function at 7–9 Years of Age in the INMA Cohort Study

**DOI:** 10.3390/ijerph191912123

**Published:** 2022-09-25

**Authors:** Laura María Compañ-Gabucio, Laura Torres-Collado, Manuela Garcia-de la Hera, Ana Fernández-Somoano, Adonina Tardón, Jordi Julvez, Jordi Sunyer, Marisa Rebagliato, Mario Murcia, Jesús Ibarluzea, Loreto Santa-Marina, Jesús Vioque

**Affiliations:** 1 Unidad de Epidemiología de la Nutrición (EPINUT), Departamento de Salud Pública, Historia de la Ciencia y Ginecología, Universidad Miguel Hernández (UMH), 03550 Alicante, Spain; 2 Instituto de Investigación Sanitaria y Biomédica de Alicante (ISABIAL), 03010 Alicante, Spain; 3 CIBER Epidemiología y Salud Pública (CIBERESP), Instituto de Salud Carlos III, 28034 Madrid, Spain; 4 Instituto Universitario de Oncología Del Principado de Asturias (IUOPA), Departamento de Medicina, Universidad de Oviedo, 33006 Oviedo, Spain; 5 Instituto de Investigación Sanitaria del Principado de Asturias (ISPA), Roma Avenue s/n, 33001 Oviedo, Spain; 6 Institut d’Investigació Sanitària Pere Virgili (IISPV), Hospital Universitari Sant Joan de Reus, 43204 Reus, Spain; 7 ISGlobal, 08003 Barcelona, Spain; 8 Department of Experimental and Health Sciences, Universitat Pompeu Fabra, 08003 Barcelona, Spain; 9 Hospital del Mar Medical Research Institute (IMIM), 08003 Barcelona, Spain; 10 Epidemiology and Environmental Health Joint Research Unit, Foundation for the Promotion of Health and Biomedical Research in the Valencian Region (FISABIO), FISABIO—Public Health, FISABIO—Universitat Jaume I-Universitat de València, 46015 Valencia, Spain; 11 Department of Medicine, Universitat Jaume I, 12071 Castellon de la Plana, Spain; 12 Servicio de Análisis de Sistemas de Información Sanitaria, Conselleria de Sanitat, Generalitat Valenciana, 46010 Valencia, Spain; 13 Group of Environmental Epidemiology, Biodonostia Health Research Institute, 20014 Donostia-San Sebastian, Spain; 14 Ministry of Health of the Basque Government, Sub-Directorate for Public Health and Addictions of Gipuzkoa, 20013 Donostia-San Sebastian, Spain; 15 School of Psychology, University of the Basque Country UPV/EHU, 20018 Donostia-San Sebastian, Spain

**Keywords:** folic acid, deficiency, high, birth cohort study, working memory, attentional function, sex specific

## Abstract

This study investigated the association between maternal low (<400 μg/day) or high (≥1000 μg/day) folic acid supplements (FAs) use during pregnancy and the attentional function and working memory in boys and girls at age 7–9. A longitudinal analysis based on 1609 mother–child pairs from the Spanish Infancia y Medio Ambiente Project was carried out. Multivariable regression analyses revealed that, compared to the recommended FAs use, a low FAs use during the second period of pregnancy was associated with a lower alertness in all children (β = 18.70 ms; 95% CI: 7.51; 29.89) and in girls (β = 30.01 ms; 95% CI: 12.96; 47.01), and with a lower N-back Task performance in boys (d’ number 2-back (β = −0.25; 95% CI: −0.49; 0.01)). A high FAs use throughout the two periods of pregnancy was associated with a better N-back Task performance only in girls (d’ number 2-back (β = 0.28; 95% CI: 0.01; 0.56) and d’ number 3-back (β = 0.32; 95% CI: 0.08; 0.56)). The maternal use of FAs beyond the periconceptional period may affect children’s attentional function and working memory at age 7–9 differently for boys and girls.

## 1. Introduction

Folic acid use is widespread worldwide due to its preventive role against the occurrence [1] and recurrence [2] of neural tube defects. It is an essential micronutrient in various neurophysiological processes during embryonic neurodevelopment, such as DNA methylation, neurotransmitter synthesis, myelination, and nucleotide synthesis [3,4,5,6]. Specifically, it plays an important role in a complex metabolic pathway closely related to optimal embryonic brain development, one-carbon metabolism [6]. Alterations in this pathway, such as a folic acid deficiency, can affect the methylation processes of neural cells in the developing brain, leading to cognitive alterations and neurodevelopmental disorders [7], such as those of childhood behavior and autism [8,9,10]. Regarding these mechanisms, a recently published systematic review [8] has suggested that optimal maternal folic acid levels during pregnancy may increase fetal brain growth and fetal brain size, which may act as a preventive factor against atypical head growth trajectories, which are common in autism.

It is recommended that mothers planning to become pregnant should take 400 μg/day of folic acid supplements (FAs) during the periconceptional period and should not exceed the tolerable upper limit of 1000 μg/day of FAs [2,11]. Although the periconceptional use of FAs has a proven beneficial effect on the health of offspring, mothers in different countries have reported noncompliance with these recommendations. Studies carried out in Poland [12] and the UK [13] showed that 42.8 and 55% of mothers did not use periconceptional FAs. In contrast, a recently published study showed that only 14% of Italian mothers did not take periconceptional FAs, but 27.5% took 5000 μg/day or above, exceeding the tolerable upper limit [14]. Similarly, it has been shown in a previous study that 55.8% of Spanish mothers used less than 400 μg/day of FAs and 29.2% exceeded 1000 μg/day [15].

These high or low periconceptional FAs doses can cause changes in the fetal brain that can lead to cognitive alterations and neuropsychological disorders in children. Thus, it has been reported that children whose mothers used periconceptional FAs doses of <400 μg/day were more susceptible to autism [9], attentional dysfunction [15], or had poorer cognitive function [10]. On the other hand, it has also been reported that children whose mothers used ≥1000 μg/day of FAs presented a psychomotor delay at 1 year of age (y) [16] and verbal delay [17] and poorer attentional function at 4–5 y [15]. As most of these studies have focused their investigations on the periconceptional period, less is known about the effect of the maternal use of FAs after the first trimester on the offspring’s cognitive function.

Mid–late pregnancy is characterized by the rapid growth and development of the fetal brain [18] and an intensive myelination [19]. These neurological processes are especially sensitive to folate concentrations [20] and are essential for children’s cognitive development [21]. Although the scientific literature in this regard is scarce, results from the randomized trial, Folic Acid Supplementation in the Second and Third Trimesters (FASSTT) [22], have shown that children whose mothers continued using 400 μg/day of FAs after the first trimester had better cognitive function at age 3 [23], improved verbal reasoning at age 7 [23], and a higher information processing speed at age 11 [24]. Interestingly, the most recent publication by FASSTT showed an improvement in girls’ verbal comprehension, suggesting a sex-specific effect on children’s cognitive function of continued FAs use of 400 μg/day beyond the first trimester [24].

In our previous study [15], not only were different effects of FAs use found on cognitive function at 4–5 y according to the sex of the children but also according to the period of pregnancy studied. For periconceptional FAs use, a detrimental effect of both <400 and ≥1000 μg/day FAs doses on attentional function was observed in boys, whereas a protective effect of <400 μg/day FAs use during the second–third trimester of pregnancy was observed in girls. Thus, in the present study, we aimed to evaluate the association between low (<400 μg/day) or high (≥1000 μg/day) FAs use in each pregnancy period (the periconceptional period, second period, and entire pregnancy) and the cognitive function in boys and girls at 7–9 y. We hypothesized that the use of both low and high doses of FAs during pregnancy can have negative effects on the attentional function and working memory of boys and girls at 7–9 y.

## 2. Materials and Methods

### 2.1. Study Desing and Population

The Infancia y Medio Ambiente (Environment and Childhood) Project (INMA; http://www.proyectoinma.org, accessed on 5 January 2022) is a Spanish multicenter mother–child prospective cohort study, whose main aim is to evaluate the effects of environmental exposures and diet during pregnancy on fetal and child development. The INMA Project is well-established in seven regions of Spain, following a common protocol [25]. In the present study, we analyzed the available information from four INMA sub-cohorts set up between 2003 and 2008 in Valencia, Sabadell (Catalonia), Asturias, and Gipuzkoa (Basque country).

**Figure 1:**
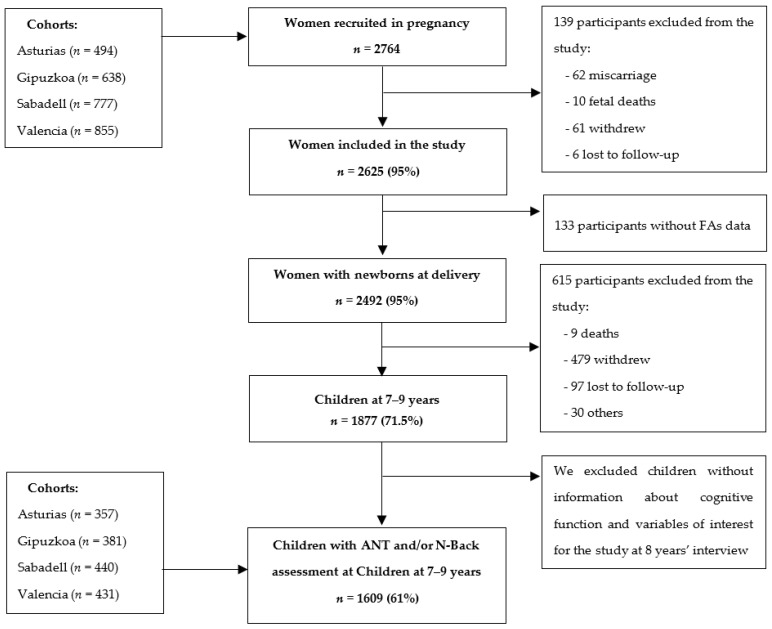
Flowchart of the study population in the main phases of the INMA project.

In the INMA cohorts, pregnant women received information about the project in their first prenatal visit at public health centers or public hospitals. Those interested in participating were recruited if they met the following criteria: (1) to be 16 years old or above, (2) to have no communication difficulties in Spanish or in another official Autonomous Community language (Basque or Catalan), (3) to have a singleton pregnancy, (4) to plan to give birth at the cohort’s referral hospital, and (5) that the pregnancy was not the result of assisted reproduction. Once pregnant women had signed the informed consent, data collection was carried out in different follow-up visits: in the first trimester of pregnancy, in the third trimester, and at delivery. After delivery, mothers and children were followed up when children were 14 months, 26 months, 4–5 y, and 7–9 y. The initial study population consisted of 2764 pregnant women, 2625 of whom gave birth to a live newborn between May 2004 and August 2008. Withdrawal from the project, being untraceable during follow-up visits, miscarriage, abortion, or fetal deaths led to the exclusion of the remaining 139 mothers. As the present study aimed to investigate FAs use during pregnancy, we excluded 133 additional mothers without FAs information. Finally, our study population consisted of 1609 mother–child pairs with complete information on the main variables when children were aged 7–9 y. In Figure 1, we present the flowchart of the study participants.

The INMA Project was approved by the Institutional Ethical Committees of the participating hospitals of each cohort (CEIC-Hospital La Fe, Valencia; CEIC-Hospital de Zumárraga, Gipuzkoa; CEIC-Parc de Salut Mar, Barcelona; CEIC-Hospital Universitario Central de Asturias). Further details on the study sub-cohorts’ characteristics, study population recruitment, and study design can be found elsewhere [25,26].

### 2.2. FAs Assessment

The assessment of FAs use during pregnancy has been described previously [16,27]. Trained personnel administered two validated food frequency questionnaires during pregnancy (FFQs) [28] to estimate pregnant women’s FAs use and dietary folate intake. The first FFQ was collected at weeks 10–13 of pregnancy to estimate FAs use and dietary folate intake from three months preconception to the third month of pregnancy, and the second FFQ was collected at weeks 28–32 to estimate FAs use and dietary folate intake from the fourth to the seventh month of pregnancy. The folate content of food was mainly obtained from USDA food composition tables [29] and other Spanish-published sources [30]. The consumption of FAs or vitamin and mineral preparations containing FAs were obtained through specific questions about supplements in the FFQ. FAs use was estimated based on daily dose, supplement brand name and composition, and duration of consumption for each period of pregnancy. As in previous studies [15,17], we estimated the average daily use of FAs and dietary folate intake for each woman in the periconceptional period (average from 3 months before pregnancy to month 3 of pregnancy), the second period of pregnancy (average from month 4 to 7), and during the whole pregnancy (average of the periconceptional and the second period). We categorized the use of FAs in the above-mentioned pregnancy periods as <400 µg/day, 400–999 µg/day (the reference category in our statistical analyses), and ≥1000 µg/day. Dietary folate intake per 100 μg/d increase was used as a covariate in adjusted models.

### 2.3. Working Memory Assessment in Children

Children’s working memory was assessed using the N-back task [31,32]. This is a continuous computerized performance task to evaluate working memory in children in approximately 25 min. For the present study, it was administered to children during the 7–9-year interview by trained personnel. During the task, children have to press a button on the keyboard if the stimulus (in this study, numbers) presented on the screen is the same as the stimulus presented earlier. The children’s responses depended on the load or level they were doing (1-back, 2-back, or 3-back). For instance, with a 1-back load, the child has to remember the previous number; in a 2-back load, the child has to remember the penultimate number; and in a 3-back load, the child has to remember the antepenultimate number presented. The 1-back load is the simplest one and, in this study, we chose to discard its results due to a ceiling effect, i.e., a perfect score was achieved by most of the children. The 2-back load can be an indicator of general mental abilities, while the highest load, the 3-back, can be an indicator of superior mental functions [33]. N-back task is used to determine detectability (d′) for each load, which was calculated as follows: d′ = z(hit rate) − z(false alarm rate). A d′ score reflects the ability to distinguish signal from noise, so a higher d′ indicates a better working memory performance [34].

### 2.4. Attentional Function Assessment in Children

Children’s attentional function was assessed with the Attentional Network Test (ANT), whose validity has been supported using neuroimaging in different studies with large child cohorts [32,35,36]. This computer test characterizes attentional function in children older than 6 y [35]. During the test, a row of five yellow fish appears either above or below a fixation point. Children are asked to “feed” the fish in the middle as quickly as possible by pressing either the right or left button on the mouse, depending on the direction in which the fish in the middle is pointing. They have to ignore the flanking fish, which point either in the same (congruent) or opposite (incongruent) direction as the fish in the middle. When children performed the test correctly, they received positive auditory reinforcement (Woo hoo!) [37]. The ANT was administered by trained personnel in the 7–9-year interview. All the children received the same instructions, and in each cohort, the same computer was used for all the children.

This test is a widely used neuropsychological tool which provides different outcomes to characterize different aspects of attentional function. First, we analyzed the three attention networks: alerting, which reflects the ability to produce and maintain optimal vigilance and performance during tasks; orienting, which involves shifting attention to sensory stimuli; and conflict, which involves detecting and resolving conflict among responses, error detection, and response inhibition. We then analyzed the number of omission errors (a measure of selective attention), the number of commission errors (a measure of impulsivity), the standard error of the hit reaction time (HRT-SE) (a measure of speed response variability), and accuracy (a measure of performance precision). Higher scores in all the above-mentioned outcomes indicate a poorer attentional function, except in the case of accuracy, for which higher scores indicate optimal performance [32,37,38,39].

### 2.5. Other Variables

Information on other sociodemographic and lifestyle characteristics was collected using structured questionnaires administered by trained field workers. The following sociodemographic and lifestyle variables regarding the mothers were used in adjusted models as potential confounders: age (in years), energy intake (in kilocalories/day), social class (I + II (high), III (medium), IV + V (low)), educational level (primary or less, secondary, university), parity (0 or ≥1), overall tobacco exposure (yes/no), which includes mothers’ smoking habit and passive exposure to tobacco during pregnancy, and pre-pregnancy BMI (continuous). In addition, we adjusted for father’s BMI (continuous) and for other children’s variables, such as sex (male, female) and age at cognitive examination (in years). Furthermore, we used the following variables in sensitivity analyses: mother’s iodine supplementation during pregnancy (<100, 100–149, ≥150 µg/day), mother’s fish intake during pregnancy (in grams/day), mother’s verbal reasoning (continuous), time of initiating FAs usage (month 0, month 1–2, month 3, no use), mother’s acetaminophen use during pregnancy (no/yes), mother’s NO_2_ exposure during pregnancy (µg/m^3^), duration of breastfeeding (no, <20 weeks, >20 weeks), and children’s dietary folate intake (in µg/day adjusted by energy intake in kilocalories).

### 2.6. Statistical Analysis

We conducted descriptive, multivariable, and sensitivity analyses using the R 3.4.2 software (R Foundation for Statistical Computing, Vienna, Austria). We performed a descriptive analysis to characterize sociodemographic, lifestyle, and obstetric factors of pregnant women from the four INMA sub-cohorts. The Kolmogorov test was used to check the normal distribution of the quantitative variables, which were all found to be non-normal. As a result, we used the non-parametric Kruskal–Wallis test for quantitative variables and the Chi-square test for qualitative variables in the bivariate descriptive analyses.

In multivariable analyses, we adjusted for those covariates with *p* value < 0.20 in bivariate analysis and those that changed the magnitude of the main effects by >10%. We excluded from the analyses those children whose score of correct responses was lower than 70%, which is considered to be a low accuracy score in the ANT test [39]. We also excluded those children whose reaction times were shorter than 200 ms because of physiological implausibility, which suggested perseverative or anticipatory responses [40]. Multiple robust linear regression models using an MM-type estimator were conducted to evaluate the association between FAs use and ANT outcomes (alerting, orienting, conflict, HRT-SE) for each pregnancy period: periconceptional period, second period of pregnancy, and throughout the entire pregnancy [41]. Negative binomial regression models were used to assess associations between FAs use and ANT count outcomes (number of omission and commission errors) for each period of pregnancy [42]. The regression coefficients of negative binomial models were exponentiated to obtain IRR (incidence rate ratios) which should be interpreted as relative risk. Multivariable censored regression (tobit) was used to estimate the association between FAs use and N-back task outcomes (detectability *n*−2 and *n*−3) as well as ANT outcome (accuracy). In addition, we used a meta-analysis to obtain the combined estimates for the four INMA sub-cohorts. The I^2^ statistic was used to quantify heterogeneity, the random effects model was applied when heterogeneity was detected (I^2^ > 50%), and when it was not detected, the fixed effects models was applied (I^2^ ≤ 50%) [43]. Because previous studies have shown that neurodevelopment during the embryonic and fetal periods may be different for girls and boys [44,45], we additionally ran models stratifying by the children’s sex.

Finally, sensitivity analyses were performed to assess the robustness of the main results. We adjusted the basal model by mother or child variables that could be related to child cognitive function development. In the basal model, we included maternal variables, such as iodine intake from supplements; fish consumption during pregnancy; verbal reasoning outcome of the similarities subtest from the Wechsler Adult Intelligence Scale-III; FAs use initiation; NO_2_ exposition during pregnancy; duration of breastfeeding; and use of acetaminophen during pregnancy. Regarding child variables, we adjusted the basal model with child dietary folate intake at age 8, assessed by validated FFQ [46]. In addition, to evaluate any potential influence on the association found, we excluded some mothers and/or children from the analysis with conditions such as preterm deliveries (*n* = 1527), mothers with medical conditions that may have influenced the use of FAs (diabetes mellitus, epilepsy, or thyroid disease) (*n* = 1318), mothers who did not use FAs during pregnancy (*n* = 1499), obese children according to the WHO criteria (97th percentile) (*n* = 1329), children with psychomotor delay (<85th percentile) at age 1 determined by the Bayley Scales of Infant and Toddler development (BSID) (*n* = 1434), children with low scores (<76.3 score) in executive function at age 5 determined by the McCarthy Scales of Children’s Abilities (MSCA) (*n* = 1283), and children with inattention at age 5, assessed by the Conner’s Kiddie Continuous Performance test (*n* = 1087).

## 3. Results

### 3.1. Study Population Characteristics

We show the general characteristics of the pregnant women and their children according to the four areas of the study in Table 1.

The median age of the mothers at delivery was 31 years old. A high proportion of the mothers attained secondary studies (41.7%), had a low social class (46.8%), and reported exposure to tobacco during pregnancy (58.1%).

During the periconceptional period of pregnancy, 14.5% of the mothers used the recommended FAs doses of 400–999 µg/day, while 54.3% used doses of <400 µg/day FAs and 31.2% used ≥1000 µg/day (Table 1). In this period of pregnancy, Sabadell was the area of study with the highest number of mothers taking doses of <400 µg/day FAs (65.2%), whereas Gipuzkoa was the area with the highest number of mothers using ≥1000 µg/day FAs doses (39.6%) (*p*-value < 0.001).

During the second period of pregnancy, the proportion of mothers using <400 µg/day FAs doses increased slightly (56.4%), while the number of mothers using ≥1000 µg/day FAs doses decreased by almost half (17.2%) (Table 1). In this period of pregnancy, Sabadell continued to be the area with the highest proportion of mothers using <400 µg/day FAs doses (83.2%), and Asturias was the area with the highest number of mothers using FAs doses of ≥1000 µg/day (34.7%) (*p*-value < 0.001).

The percentage of boys and girls participating in this study was almost the same, 50.7 and 49.3%, respectively (Table 1). The median age of the children at the time of the neurocognitive assessment was 7.7 years old, but it was higher in the children from Asturias (8.3 years) and lower in the children from Sabadell (6.8 years) (*p*-value < 0.001). Regarding the ANT outcomes (related to attentional function), the children from Gipuzkoa scored higher in the conflict attention network (78.0 ms; 36.0–128.0) (*p*-value = 0.004) which means a lower capacity to detect and resolve changes in attention to sensory stimuli. However, according to other ANT outcomes, the children from Valencia appeared to have the worst attentional performance compared to the other areas of study. These children scored higher in the HRT-SE (323.2 ms; 266.0–382.3) which reflects a higher response variability throughout the test (*p*-value < 0.001), that is, a poorer sustained attention. Furthermore, they scored higher in the omission (3; 1–7) and commission (4; 2–7) errors (*p*-value < 0.001), suggesting inattentiveness and impulsivity, respectively. Regarding the N-back task outcomes (related to working memory), almost all the children achieved the highest score in the d’ numbers 1-back (3.9; 3.4–3.9). Finally, the children from Asturias obtained the highest results (i.e., better performance) in the d’ numbers 2-back (2.3; 1.5–3.9) and in the d’ numbers 3-back (1.4; 1.0–2.2) together with children from Gipuzkoa (1.4; 1.0–1.9) (*p*-value < 0.001).

### 3.2. FAs Use during Pregnancy and Cognitive Outcomes in Children

The fully adjusted models with the combined associations between the maternal FAs use in each period of pregnancy and the scores in the neurocognitive tests in the children at age 7–9 are shown in Table 2.

**Figure 2:**
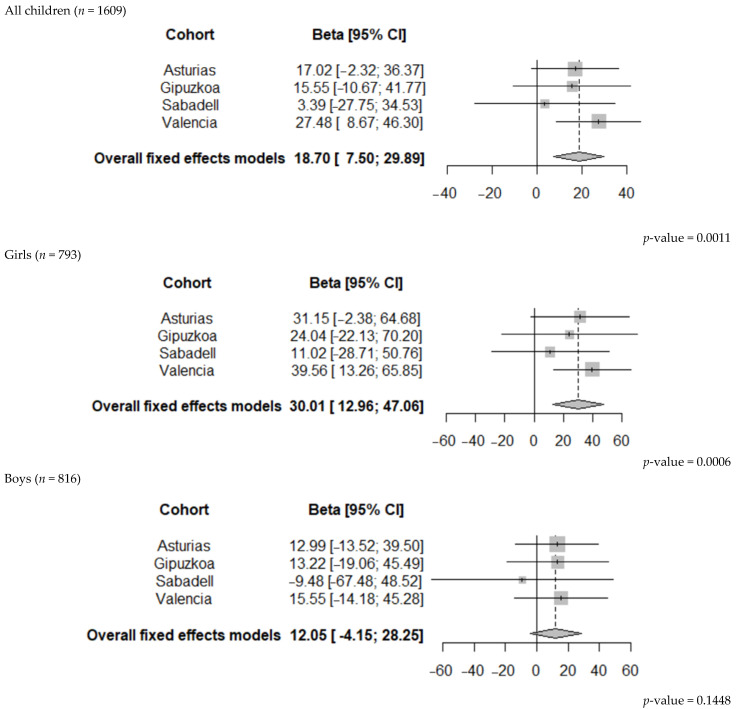
Pooled estimates of the associations between FAs (<400 µg/day compared to 400–999 µg/day) in the second period and alerting in all children, boys, and girls at 7–9 years of age.

Compared to 400–999 µg/day, we observed a statistically significant association between the use of FAs < 400 µg/day in the second period of pregnancy and a higher alerting score in the children at age 7–9 (β = 18.70, 95% CI 7.51; 29.89). In other words, the offspring of mothers who used lower doses of FAs (<400 µg/day) than recommended during the second period of pregnancy were less able to achieve and maintain an optimal alertness. This negative association was similar in each cohort of the study, although in Valencia, it was stronger and reached a statistical significance (Figure 2).

### 3.3. FAs Use during Pregnancy and Cognitive Outcomes in Children by Sex

The associations between the maternal FAs use in each period of pregnancy and the cognitive outcomes at 7–9 y are shown in Table 3 for the girls and in Table 4 for the boys.

When the analyses were stratified by sex, no statistically significant associations were found between the maternal use of FAs during the periconceptional period and the cognitive outcomes (ANT—attention—and N-back task—working memory) for the girls (Table 3) or for the boys (Table 4).

During the second period of pregnancy, we found a detrimental effect of the maternal FAs use of <400 µg/day, compared to the recommended use of FAs (400–999 µg/day), on the girls’ alerting (ANT outcome) (β = 30.01, 95% CI 12.96; 47.01) (Table 3), but this effect was not statistically significant in the boys (β = 12.05, 95% CI −4.15; 28.25) (Table 4). In the boys, we observed a statistically significant association between the maternal use of FAs below the recommended dose (<400 µg/day) and working memory (N-back task). Specifically, the boys whose mothers used <400 µg/day FAs doses compared to those who used 400–999 µg/day performed worse in the d’ numbers 2-back (β = −0.25, 95% CI −0.49; 0.01) (Table 4). We found no other statistically significant associations in either the girls or boys.

Throughout pregnancy, compared to the recommended use of FAs (400–999 µg/day), we observed a statistically significant association between ≥ 1000 µg/day maternal FAs use and girls’ attentional function (ANT). Specifically, the girls whose mothers used higher than recommended doses of FAs (≥1000 µg/day) had a lower HRT-SE score (β = −20.38, 95% CI −39.12; −1.63), which indicates better sustained attention (Table 3). These FAs doses were also associated with an improved working memory performance (N-back task) in the girls, particularly regarding both the d’ numbers 2-back and d’ numbers 3-back (β = 0.28, 95% CI 0.01; 0.56 and β = 0.32, 95% CI 0.08; 0.56, respectively) (Table 3). No statistically significant associations were found between the maternal use of FAs and the attentional function or working memory for the boys in this period of pregnancy (Table 4).

### 3.4. Sensitivity Analyses

**Figure 3:**
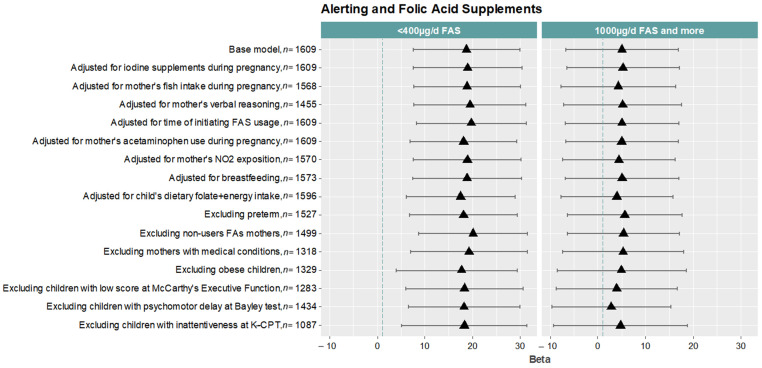
Sensitivity analyses of the associations between FAs use (<400 and ≥1000 compared to 400–999 µg/day) during the second period of pregnancy and alerting in all children (*n* = 1609) at age 7–9.

We carried out several sensitivity analyses under different scenarios to assess the robustness of the association found between FAs use in the second period of pregnancy (<400 vs. 400–999 µg/day) and the children’s alerting (Figure 3).

Overall, the associations remained similar to the basal model (β = 18.70, 95% CI 7.51; 29.89). We observed a slightly stronger association when we adjusted for some mothers’ conditions, such as the initiation of FAs use (β = 19.71, 95% CI 8.20; 31.22), the non-use of FAs in any period of pregnancy (β = 20.11, 95% CI 8.68; 31.54), and verbal reasoning (β = 19.45, 95% CI 7.70; 31.19) and also when we excluded mothers with medical conditions (β = 19.24, 95% CI 7.03; 31.46). In contrast, the association became slightly weaker when we adjusted for the dietary folate intake of the children at age 8 (β = 17.48, 95% CI 6.12; 28.84) and when we excluded those children with obesity (β = 16.67, 95% CI 4.04; 29.31) (Figure 3).

## 4. Discussion

In this study, we found that the children of mothers using less than 400 µg/day of FAs during the second period of pregnancy presented more impairment in attentional function (i.e., alertness) at the age of 7–9 y than the children of mothers using 400–999 µg/day. We also found that the girls whose mothers used <400 µg/day FAs had a poorer attentional function (i.e., alertness) at the age of 7–9 y, while the boys showed a poorer working memory. Moreover, the girls whose mothers used ≥1000 µg/day FAs during the entire pregnancy showed a better sustained attention and working memory at 7–9 y. Finally, contrary to our expectations, we did not find associations between periconceptional FAs use and children’s neurocognitive functions.

It is well known that the use of recommended doses of FAs (400 µg/day) during the periconceptional period can prevent abnormalities in fetal central nervous system development, such as neural tube defects [47]. A growing number of studies have shown that the use of recommended periconceptional FAs doses is related to improvements in the neurocognitive functioning in childhood [48]. Conversely, a deficient periconceptional FAs use has been associated with a harmful effect on mental development at age 1 [49], while a high FAs use may be related to a psychomotor delay at age 1 [16] and verbal deficits at age 5 [17]. In a previous study, when mothers who used recommended periconceptional FAs doses were compared to those who used non-recommended doses, a negative association between FAs use of <400 and ≥1000 µg/day and children’s attentional function at age 5 was observed [15]. The use of a similar population of study led us to hypothesize a potential association between non-recommended periconceptional FAs use and the attentional function and/or working memory of children at age 7–9, but contrary to our expectations, we did not find any association. We do not have a clear explanation for this lack of association, although it could be due in part to the difference in age of the children in the two studies and thus to a different level of brain maturation and cognitive function development [35,50,51].

We found a reduced alertness in children of 7–9 y whose mothers used low doses of FAs during the second period of pregnancy. The use of FAs during this period of pregnancy has not been sufficiently studied because recommendations for its use are focused on the periconceptional period. Nevertheless, the results on the use of FAs in this second period reported by the Folic Acid Supplementation in the Second and Third Trimesters (FASSTT trial) randomized trial support our findings [22]. To our knowledge, this is the only randomized trial which analyzed the effects of continuing to take 400 µg/day of FAs after the first trimester of pregnancy. In this trial, before the second period of pregnancy began, 119 mothers who took 400 µg/day during the first trimester of pregnancy were randomized into two groups, placebo or continuing to take 400 µg/day. They found that children whose mothers continued to take 400 µg/day scored higher in word reasoning at age 7 [23] and cognition at ages 3 [23] and 11 [24]. Similarly, an observational study has associated a deficient maternal folate status after the first trimester with detrimental effects on children’s neurocognitive functions, such as reduced brain volume at 6–8 y and lower language and visuospatial abilities [10]. This could be because the second period of pregnancy is characterized by the rapid growth and myelinization of the fetal brain, which are processes closely related to optimal cognitive functioning and are particularly sensitive to maternal folate deficiency [19,20,21].

We also found that the association between maternal FAs use beyond the first trimester and children’s cognitive functions at 7–9 y was different in girls and boys. We observed that a low use of FAs during the second period of pregnancy was associated with a poorer alertness in girls and a poorer working memory in boys. In contrast, a previous study [15] showed that girls at 4–5 y whose mothers took low FAs doses after the first trimester presented higher attentiveness, although an association in boys was found. The discrepancies between the results of the two studies can be explained by the children’s age difference and, thus, the different development levels of their cognitive functions. The attentional function and working memory undergo important development during early childhood [52], especially at age 7–9 [53,54]. This age is characterized by a pronounced development of the brain frontal lobe [35], a key structure which contributes to the improvement of both these high cognitive functions [36]. In particular, during this stage, girls perform more accurately in tasks related to the working memory [55] and attentional function than boys [37], suggesting that girls have a more developed cognitive function than boys at the same age. This fact may partly explain the different effect of maternal FAs on the cognitive function of girls and boys.

To our knowledge, no other previous studies have specifically analyzed the effect of the maternal use of FAs after the first trimester of pregnancy on children’s attention and/or working memory. However, in line with our present results, some studies have shown different sex-specific effects of maternal FAs use on other cognitive functions, such as verbal function. For instance, Caffrey et al. [24] found a beneficial effect of 400 µg/day FAs use beyond the first trimester on verbal comprehension in girls at age 11, but not in boys. Similarly, an observational study carried out in the Indian population found that the association between an adequate maternal folate status at week 30 of pregnancy with verbal fluency was stronger in girls than boys at 9–10 y [56]. One explanation for these findings could be the sex-specific DNA methylation response to FAs use after the first trimester. In this regard, a previous study has shown that the DNA methylation in genes related to brain development appears to be different in boys and girls [57]. Another possible explanation could be the sex differences in brain activity patterns related to cognition and functional brain development in infancy, childhood [19], and even in adolescence [39]. Girls present a higher rate of myelination than boys in some brain structures between 3 and 60 months, such as the corpus callosum and left frontal and left temporal white matter [58]. At 7–9 y, a rapid growth takes place in the connections of the frontal lobe, a brain structure which is closely related to working memory and attentional function [53,54]. During this period, a more pronounced cognitive growth was documented in girls [55]. Specifically, girls appeared to experience earlier working memory maturation peaks [59,60] and more advanced attentional function [37], suggesting a biological basis for the differences found between boys and girls.

We also found that the use of high doses of FAs (≥1000 µg/day) throughout pregnancy improved girls’ sustained attention and working memory at 7–9 y. High FAs use may result in the presence of unmetabolized folic acid in the bloodstream that can saturate our body mechanism to convert folic acid to folate [61], leading to a reduced involvement of folate in fetal neurodevelopmental processes. For this reason, we expected to find a negative effect, if any, of high FAs use during pregnancy on children’s cognitive function. This can be justified in two ways. First, girls have been described as presenting better sustained attention than their male peers [62]. Second, the effect of high FAs use during pregnancy on the health of offspring is still inconclusive [61]. The maternal use of high periconceptional doses of FAs has been associated with a higher risk of newborns being small for gestational age for both weight and height at birth [63,64]. In contrast, the maternal use of high FAs doses during pregnancy did not appear to increase the risk of neural tube defects [65] or preterm births [66]. In addition, high FAs doses have been associated with a reduced risk of congenital heart problems [67], low weight at birth [66], and congenital male genital abnormalities at birth [68]. With regard to cognitive function, high FAs use has been associated with poorer attention at 4–5 y in both boys and girls [15] but also with an improved vocabulary at 18 months [69]. We have no explanation for this unexpected finding, although this is not the first time that a protective effect on children’s cognitive function of an apparently harmful exposure during pregnancy has been described in the scientific literature. A multicentric birth cohort study in six European countries has associated higher prenatal mercury levels and maternal alcohol consumption, two harmful exposures, during pregnancy with better cognitive performance in children at 6–11 y [70]. Similarly, another study has shown that higher organochlorine prenatal exposure can increase attentional function (i.e., the hit reaction time) in girls at age 8 [71]. Nevertheless, we should also be aware that all the above unexpected results may be due to reverse causation or residual confounding. For instance, one possible outcome related to high FAs use, and thus to residual confounding, is maternal illness. Pregnant women who take high doses of FAs during pregnancy usually do so because they have an illness which can reduce the absorption of folic acid. This is the case for epilepsy, whose treatment acts as a folic acid antagonist, thus making a high dose of FAs necessary to prevent neural tube defects in offspring [72]. However, in our study population, when we performed the sensitivity analyses excluding mothers with health conditions that could influence the associations found, the results did not change.

Our findings can also be explained by the involvement of folic acid in epigenetic mechanisms during pregnancy. Inadequate maternal nutrition can disrupt the fetal epigenetic mechanisms [73], which can lead to adverse postnatal health outcomes, such as neurodevelopmental disorders [74]. Folic acid is involved in a complex pathway called one-carbon metabolism, which is responsible for providing a number of substrates for fetal epigenetic processes [75]. In addition, one-carbon metabolism appears to control the fetal brain cell population and brain epigenetic expression through DNA methylation [6]. Thus, alterations in the one-carbon metabolism, especially folate deficiency, could be associated with neurodevelopmental disorders and behavioral and cognitive problems in childhood [7,9]. However, the role of one-carbon metabolism in brain development and the long-term cognitive effects are still mostly unknown.

In brief, our findings provide new evidence on the effects of the use of non-recommended FAs doses during different periods of pregnancy on cognitive function in children at age 7–9. Although our results are not confirmatory enough and they need to be confirmed, they may help to establish new recommendations for the use of FAs in the Spanish population, with a special focus on mid–late pregnancy, as the recommended FAs use during this period can be beneficial to cognitive development in children. In addition, our results suggest that girls are more susceptible than boys to non-recommended doses of FAs during pregnancy, although this is a subject of study that requires further investigation.

This study presents some limitations. First, it has an observational design, and the risk of uncontrolled or residual confounding exists. Although we have adjusted the models for a wide variety of potential risk factors and have also conducted sensitivity analyses, it is possible that we have overlooked some potentially confounding variables. In this sense, we have not taken into account the variation between neurocognitive assessors as a possible confounder variable, although all the assessors were trained to conduct the evaluations, so differences between them should be minimal. Second, there is a possible bias due to subjects lost to follow-up. However, the mothers of those children who performed the cognitive assessment at age 7–9 presented very similar age, educational, and socioeconomic statuses as well as FAs use compared to mothers who were excluded from the analyses because of lost to follow-up or because their children did not carry out the cognitive assessment. Third, our results should be interpreted with caution because no formal interaction by sex was observed. Fourth, maternal FAs use was calculated according to self-reported responses, which tend to be overreported in the case of healthy behaviors and underreported in the case of negative ones. Self-reported estimations can cause a misclassification, but if it does, it would likely be non-differential. In this sense, to classify mothers according to their FAs use, we used an FFQ which may be limited when estimating exact absolute values, although it was previously validated in this population of pregnant women [28]. Fifth, our results may not be generalizable to all countries due to differences in food folic acid fortification policies; it should be noted that in Spain and most of Europe, foods are not fortified. Finally, our results do not explain the neurophysiological mechanisms which lead the use of non-recommended doses of FAs during pregnancy to affect cognitive function at 7–9 y.

Several strengths should be also considered. The prospective design of the INMA project may help to minimize recall and selection bias, and although the sample size was not very large, we were able to detect significant associations from the joint efforts of four well-established INMA cohorts with common protocols. In addition, we used a validated FFQ test to estimate the maternal FAs intake during pregnancy. We also used standardized and computerized batteries to assess children’s cognitive function, which can detect small changes in children’s responses and provide objective assessments. The cognitive assessment was performed by trained personnel blinded to exposure status, representing a more unbiased assessment of the possible associations between maternal FAs use and cognitive function. We showed the detailed information on the doses of FAs use during two distinct periods of pregnancy, which few published studies have provided. Thus, the present study together with our previous study are the only ones that have examined the effect of maternal FAs use in different periods of pregnancy on children’s attentional function and working memory in the Spanish population. Finally, we carried out several sensitivity analyses which reinforced the consistency of our results, and they also provide evidence of sex differences in the effects of maternal FAs use during pregnancy, opening new lines of research.

## 5. Conclusions

This study suggests that FAs use beyond the first trimester of pregnancy at the current periconceptional recommended dose to prevent neural tube defects can improve children’s alertness at age 7–9. In addition, a low FAs use can have a different effect on girls and boys. In the case of girls, it can reduce alertness, while in boys, it can reduce working memory, two essential cognitive functions for academic and daily life performance. Thus, although the existing recommendations for FAs use focus on the periconceptional period of pregnancy, our findings may add evidence to support the need to continue taking the recommended FAs dose of 400 µg/day after the first trimester of pregnancy to ensure the proper cognitive development of both girls and boys at age 7–9. Further studies in this line of research are needed to determine these recommendations.

## Figures and Tables

**Table 1:** Socio-demographic, lifestyle, and obstetric characteristics of parents and children at 7-year follow-up of INMA cohort study, Spain, 2003–2008.

	All Cohorts, *n* = 1609	Valencia, *n* = 431	Sabadell, *n* = 440	Asturias, *n* = 357	Gipuzkoa, *n* = 381	*p*-Value ^1^
Mother’s age, y	31 (28; 34)	30 (28; 33)	30 (28; 33)	32 (29; 35)	31 (29; 33)	<0.001
Mother’s education						<0.001
Primary or less	300 (18.7)	111 (25.8)	102 (23.2)	48 (13.4)	39 (10.2)	
Secondary	671 (41.7)	186 (43.2)	191 (43.4)	160 (44.8)	134 (35.2)	
University	638 (39.7)	134 (31.1)	147 (33.4)	149 (41.7)	208 (54.6)	
Mother’s social class						<0.001
I + II (high)	406 (25.2)	89 (20.6)	100 (22.7)	88 (24.6)	129 (33.9)	
III	450 (28.0)	117 (27.1)	144 (32.7)	76 (21.3)	113 (29.7)	
IV + V (low)	753 (46.8)	225 (52.2)	196 (44.5)	193 (54.1)	139 (36.5)	
Parity ≥ 1	680 (42.3)	189 (43.9)	188 (42.7)	137 (38.4)	166 (43.6)	0.398
Overall tobacco exposition during pregnancy, yes	934 (58.1)	315 (73.1)	260 (59.1)	156 (43.7)	203 (53.3)	<0.001
Missing values	41 (2.6)	5 (1.2)	8 (1.8)	18 (5.0)	10 (2.6)	
Prepregnancy mother’s BMI, kg/m^2^						0.008
<25	1183 (73.5)	311 (72.2)	317 (72.0)	248 (69.5)	307 (80.6)	
≥25–30	311 (19.3)	85 (19.7)	84 (19.1)	84 (23.5)	58 (15.2)	
≥30	115 (7.2)	35 (8.1)	39 (8.9)	25 (7.0)	16 (4.2)	
Prepregnancy father’s BMI, kg/m^2^						<0.001
<25	687 (42.7)	187 (43.4)	204 (46.4)	114 (31.9)	182 (47.8)	
≥25–30	704 (43.6)	199 (46.2)	170 (38.6)	175 (49.0)	160 (42.0)	
≥30	189 (11.8)	45 (10.4)	60 (13.6)	55 (15.4)	29 (7.6)	
Missing values	29 (1.8)	0 (0.0)	6 (1.4)	13 (3.6)	10 (2.6)	
Dietary folate, µg/d						
In 1st period of pregnancy	297 (239; 364)	292 (231; 359)	283 (230; 347)	313 (247; 382)	306 (255; 367)	<0.001
In 2nd period of pregnancy	297 (241; 358)	274 (219; 349)	291 (237; 345)	302 (245; 367)	312 (266; 370)	<0.001
FAs µg/d, in 1st period of pregnancy						<0.001
400–999	233 (14.5)	68 (15.8)	45 (10.2)	64 (17.9)	56 (14.7)	
<400	874 (54.3)	249 (57.8)	287 (65.2)	164 (45.9)	174 (45.7)	
≥1000	502 (31.2)	114 (26.5)	108 (24.5)	129 (36.1)	151 (39.6)	
FAs µg/d, in 2nd period of pregnancy						<0.001
400–999	426 (26.5)	200 (46.4)	52 (11.8)	133 (37.3)	41 (10.8)	
<400	907 (56.4)	144 (33.4)	366 (83.2)	100 (28.0)	297 (78.0)	
≥1000	276 (17.2)	87 (20.2)	22 (5.0)	124 (34.7)	43 (11.3)	
Child’s sex						0.631
Boys	816 (50.7)	210 (48.7)	229 (52.0)	188 (52.7)	189 (49.6)	
Girls	793 (49.3)	221 (51.3)	211 (48.0)	169 (47.3)	192 (50.4)	
Child’s age ^2^, in years	7.7 (7.3; 8.0)	7.6 (7.4; 7.6)	6.8 (6.6; 7.1)	8.3 (8.1; 8.4)	7.9 (7.8; 8.0)	<0.001
ANT outcomes						
HRT-SE, ms	305.6 (245; 364)	323.2 (266; 382)	320.3 (260; 369)	260.8 (196; 329)	304.3 (259; 356)	<0.001
Accuracy	1.0 (1.0; 1.0)	1.0 (0.9; 1.0)	1.0 (0.9; 1.0)	1.0 (1.0; 1.0)	1.0 (1.0; 1.0)	<0.001
Comission errors, num	3 (1; 5)	4 (2; 7)	4 (2; 7)	1 (0; 3)	2 (1; 4)	<0.001
Omission errors, num	2 (0; 5)	3 (1; 7)	2 (1; 5)	0 (0; 2)	2 (0; 4)	<0.001
Alerting	45 (−3.5; 100.5)	52 (0.8; 101.8)	39 (−10.6; 100.9)	45 (4.0; 90.5)	48.5 (−6.0; 108.0)	0.314
Orienting	38.5 (−14.0; 85.0)	40.0 (−15.5; 88.5)	30.5 (−17.5; 81.3)	41.5 (0.0; 83.0)	38.0 (−17.0; 85.5)	0.358
Conflict, ms	71 (32.5; 115)	68.5 (28.5; 111)	75.5 (35.9; 122)	63.0 (33.5; 101)	78.0 (36.0; 128)	0.004
N-back outcomes						
d’ numbers 1-back	3.9 (3.4; 3.9)	3.9 (2.8; 3.9)	3.9 (2.6; 3.9)	3.9 (3.9; 3.9)	3.9 (3.9; 3.9)	<0.001
d’ numbers 2-back	1.9 (1.1; 2.6)	1.7 (1.0; 2.5)	1.7 (1.0; 2.3)	2.3 (1.5; 3.9)	2.0 (1.3; 2.7)	<0.001
d’ numbers 3-back	1.1 (0.6; 1.7)	1.0 (0.3; 1.7)	1.0 (0.3; 1.4)	1.4 (1.0; 2.2)	1.4 (1.0; 1.9)	<0.001

FAs, folic acid supplement; HRT-SE, hit reaction time standard error (ms); INMA, Infancia y Medio Ambiente; ms, milliseconds; num, number. Values are medians (IQRs) for mother’s age, age at cognitive examination, dietary folate, ANT outcomes and N-back outcomes, and values are *n* (%) for the rest of variables. ^1^ *p*-values of differences between study cohorts from Chi-square test (categorical variables) and Kruskal–Wallis test (continuous non-parametric variables), ^2^ age at neurocognitive examination.

**Table 2:** Fully adjusted combined association between folic acid supplements (FAs) intake during pregnancy and attentional function in children aged 7–9 y of INMA cohort study, Spain, 2003–2008.

			Periconceptional Period ^a^		Second Period ^a^			Entire Pregnancy ^a^		
Attentional Network Test (*n* = 1609)	FAS (µg/d)	*n*	β ^b^	(95% CI)	*n*	β ^b^	(95% CI)	*n*	β ^b^	(95% CI)
HRT-SE (ms) ^c^	400–999	233	Ref.		426	Ref.		299	Ref.	
	<400	874	−4.07	(−15.86; 7.73)	907	−5.02	(−16.21; 6.17)	885	−4.64	(−15.85; 6.56)
	≥1000	502	−6.52	(−19.09; 6.04)	276	−7.98 ^#^	(−29.37; 13.41)	425	−10.96	(−23.23; 1.31)
Accuracy	400–999	233	Ref.		426	Ref.		299	Ref.	
	<400	874	0.00	(−0.00; 0.00)	907	−0.00	(−0.00; 0.00)	885	−0.00	(−0.01; 0.00)
	≥1000	502	0.00	(−0.00; 0.00)	276	0.00	(−0.00; 0.01)	425	0.00	(−0.00; 0.01)
Commission errors (num) ^c^	400–999	233	Ref.		426	Ref.		299	Ref.	
	<400	874	0.94	(0.82; 1.08)	907	1.02	(0.89; 1.14)	885	1.05	(0.92; 1.19)
	≥1000	502	0.90	(0.77; 1.04)	276	1.00	(0.87; 1.16)	425	0.99	(0.86; 1.14)
Omission errors (num) ^c^	400–999	233	Ref.		426	Ref.		299	Ref.	
	<400	874	1.08	(0.90; 1.30)	907	0.96 ^#^	(0.74; 1.25)	885	0.96	(0.81; 1.14)
	≥1000	502	0.94	(0.77; 1.15)	276	0.87	(0.71; 1.06)	425	0.85	(0.70; 1.04)
Alerting ^c^	400–999	233	Ref.		426	Ref.		299	Ref.	
	<400	874	−7.83	(−21.56; 5.90)	907	18.70 *	(7.51; 29.89)	885	6.27	(−5.56; 18.11)
	≥1000	502	−8.33	(−22.52; 5.86)	276	5.03	(−6.72; 16.77)	425	−1.05	(−13.29; 11.20)
	400–999	233	Ref.		426	Ref.		299	Ref.	
Orienting ^c^	<400	874	−5.95 ^#^	(−29.52; 17.62)	907	1.03	(−9.38; 11.44)	885	2.23	(−9.03; 13.48)
	≥1000	502	−8.25 ^#^	(−27.26; 10.77)	276	−0.91	(−13.66; 11.84)	425	−0.86	(−13.29; 11.54)
	400–999	233	Ref.		426	Ref.		299	Ref.	
Conflict (ms) ^c^	<400	874	3.78	(−5.65; 13.21)	907	−0.62	(−8.99; 7.76)	885	−1.71	(−10.63; 7.21)
	≥1000	502	6.51	(−3.47; 16.49)	276	1.69	(−7.80; 11.18)	425	−2.81	(−12.57; 6.95)
**N-back task (*n* = 1312)**										
d’ number 2-back	400–999	193	Ref.		346	Ref.		238	Ref.	
	<400	703	−0.04	(−0.23; 0.14)	721	−0.14	(−0.31; 0.03)	709	0.10	(−0.08; 0.28)
	≥1000	416	−0.05	(−0.24; 0.15)	245	−0.03	(−0.22; 0.16)	365	0.15	(−0.04; 0.34)
d’ number 3-back	400–999	276	Ref.		346	Ref.		238	Ref.	
	<400	829	0.05	(−0.11; 0.21)	721	0.06	(−0.09; 0.20)	709	0.03	(−0.12; 0.19)
	≥1000	398	0.03	(−0.13; 0.20)	245	0.00	(−0.16; 0.16)	365	0.05	(−0.12; 0.21)

^a^ Models were adjusted by energy intake (in kilocalories), dietary folate intake per 100 µg/d increase, cohort, social class (I + II (high), III or IV + V (low)), educational level (primary or less, secondary, or university), parity (0 or ≥1), tobacco exposition during the periconceptional period (no or yes), mother’s age (in years), mother’s BMI (continuous), father’s BMI (continuous), child’s sex, and child’s age at 7 y follow-up examination (in years). ^b^ Robust linear regression model was used for hit reaction time and hit reaction time SE (HRT-SE). Negative binomial regression model was used for commission and omission errors (estimates are incidence rate ratios, IRR). Tobit regression model was used for N-back task outcomes and accuracy. ^c^ Higher scores means lower performance. * *p*-value < 0.05. ^#^ I^2^ > 50%, random models were used.

**Table 3:** Fully adjusted combined association between folic acid supplements (FAs) intake during pregnancy and attentional function in girls aged 7–9 y of INMA cohort study, Spain, 2003–2008.

			Periconceptional Period ^a^			Second Period ^a^		Entire Pregnancy ^a^		
Attentional Network Test (*n* = 793)	FAS (µg/d)	*n*	β ^b^	(95% CI)	*n*	β ^b^	(95% CI)	*n*	β ^b^	(95% CI)
HRT-SE (ms) ^c^	400–999	129	Ref.		216	Ref.		445	Ref.	
	<400	425	−3.33	(−18.37; 11.62)	437	−14.25	(−29.15; 0.64)	215	−15.85	(−32.61; 0.91)
	≥1000	239	−3.22 ^#^	(−30.25; 23.81)	140	−7.49 ^#^	(−42.23; 27.25)	133	−20.38 *	(−39.12; −1.63)
Accuracy	400–999	129	Ref.		216	Ref.		445	Ref.	
	<400	425	−0.00	(−0.01; 0.01)	437	0.00	(−0.00; 0.01)	215	−0.00	(−0.01; 0.00)
	≥1000	239	0.00	(−0.00; 0.01)	140	0.00	(−0.00; 0.01)	133	−0.00	(−0.01; 0.01)
Commission errors (num) ^c^	400–999	129	Ref.		216	Ref.		445	Ref.	
	<400	425	1.00 ^#^	(0.64; 1.35)	437	0.88	(0.75; 1.04)	215	0.98	(0.82; 1.18)
	≥1000	239	0.93	(0.76; 1.14)	140	0.88	(0.72; 1.08)	133	0.97	(0.79; 1.19)
Omission errors (num) ^c^	400–999	129	Ref.		216	Ref.		445	Ref.	
	<400	425	1.21	(0.95; 1.54)	437	1.03 ^#^	(0.58; 1.84)	215	0.85	(0.66; 1.09)
	≥1000	239	0.93	(0.72; 1.22)	140	0.97 ^#^	(0.46; 2.03)	133	0.75	(0.49; 1.16)
Alerting ^c^	400–999	129	Ref.		216	Ref.		445	Ref.	
	<400	425	−17.93	(−39.81; 3.95)	437	30.01	(12.96; 47.01) *	215	8.96	(−11.55; 29.48)
	≥1000	239	−16.14	(−38.53; 6.25)	140	6.23	(−10.86; 23.32)	133	−0.41	(−21.04; 20.23)
	400–999	129	Ref.		216	Ref.		445	Ref.	
Orienting ^c^	<400	425	−18.07 ^#^	(−42.59; 6.44)	437	4.37	(−11.06; 19.80)	215	3.80	(−14.72; 22.31)
	≥1000	239	−12.47	(−29.91; 4.97)	140	13.27	(−4.56; 31.10)	133	7.65	(−11.74; 27.04)
	400–999	129	Ref.		216	Ref.		445	Ref.	
Conflict (ms) ^c^	<400	425	7.88	(−5.98; 21.73)	437	1.74	(−11.32; 14.81)	215	5.06	(−9.98; 20.11)
	≥1000	239	11.60	(−3.51; 26.71)	140	1.77	(−12.15; 15.69)	133	3.70	(−12.04; 19.44)
**N-back task (*n* = 639)**										
d’ number 2-back	400–999	107	Ref.		168	Ref.		106	Ref.	
	<400	336	−0.01	(−0.25; 0.23)	343	−0.06	(−0.29; 0.18)	351	0.21	(−0.05; 0.46)
	≥1000	196	0.00	(−0.26; 0.26)	128	0.09	(−0.18; 0.36)	182	0.28 *	(0.01; 0.56)
d’ number 3-back	400–999	107	Ref.		168	Ref.		106	Ref.	
	<400	336	−0.06	(−0.27; 0.16)	343	0.14	(−0.07; 0.34)	351	0.12	(−0.11; 0.34)
	≥1000	196	0.09	(−0.14; 0.32)	128	0.14	(−0.10; 0.37)	182	0.32 *	(0.08; 0.56)

^a^ Models were adjusted by energy intake (in kilocalories), dietary folate intake per 100 µg/d increase, cohort, social class (I + II (high), III or IV + V (low)), educational level (primary or less, secondary, or university), parity (0 or ≥1), tobacco exposition during the periconceptional period (no or yes), mother’s age (in years), mother’s BMI (continuous), father’s BMI (continuous), and child’s age at 7 y follow-up examination (in years). ^b^ Robust linear regression model was used for hit reaction time and hit reaction time SE (HRT-SE). Negative binomial regression model was used for commission and omission errors (estimates are incidence rate ratios, IRR). Tobit regression model was used for N-back task outcomes and accuracy. ^c^ Higher scores means lower performance. * *p*-value < 0.05. ^#^ I^2^ > 50%, random models were used.

**Table 4:** Fully adjusted combined association between folic acid supplements (FAs) intake during pregnancy and attentional function in boys aged 7–9 y of INMA cohort study, Spain, 2003–2008.

			Periconceptional Period ^a^			Second Period ^a^		Entire Pregnancy ^a^		
Attentional Network Test (*n* = 816)	FAS (µg/d)	*n*	β ^b^	(95% CI)	*n*	β ^b^	(95% CI)	*n*	β ^b^	(95% CI)
HRT-SE (ms) ^c^	400–999	104	Ref.		210	Ref.		166	Ref.	
	<400	449	−8.92	(−27.00; 9.16)	470	7.19	(−10.05; 24.43)	440	5.15	(−10.20; 20.50)
	≥1000	263	−8.59	(−27.10; 9.92)	136	−3.20	(−23.69; 17.28)	210	−2.35	(−19.44; 14.73)
Accuracy	400–999	104	Ref.		210	Ref.		166	Ref.	
	<400	449	0.01	(−0.00; 0.01)	470	−0.00	(−0.01; 0.00)	440	0.00	(−0.00; 0.01)
	≥1000	263	0.01	(−0.00; 0.01)	136	−0.00	(−0.01; 0.01)	210	0.00	(−0.00; 0.01)
Commission errors (num) ^c^	400–999	104	Ref.		210	Ref.		166	Ref.	
	<400	449	0.88	(0.72; 1.07)	470	1.15	(0.97; 1.37)	440	1.04	(0.87; 1.23)
	≥1000	263	0.84	(0.68; 1.03)	136	1.06	(0.86; 1.29)	210	0.94	(0.78; 1.14)
Omission errors (num) ^c^	400–999	104	Ref.		210	Ref.		166	Ref.	
	<400	449	1.05	(0.79; 1.39)	470	1.02	(0.80; 1.30)	440	1.10	(0.87; 1.40)
	≥1000	263	1.05	(0.79; 1.41)	136	0.99	(0.74; 1.31)	210	1.00 ^#^	(0.61; 1.61)
Alerting ^c^	400–999	104	Ref.		210	Ref.		166	Ref.	
	<400	449	2.08	(−16.12; 20.28)	470	12.05	(−4.15; 28.25)	440	2.75	(−12.31; 17.81)
	≥1000	263	1.16	(−18.37; 20.70)	136	7.84	(−9.81; 25.49)	210	0.52	(−15.97; 17.02)
	400–999	104	Ref.		210	Ref.		166	Ref.	
Orienting ^c^	<400	449	9.27	(−7.58; 26.12)	470	−0.20	(−15.00; 14.61)	440	1.12 ^#^	(−23.82; 26.05)
	≥1000	263	−1.66	(−19.91; 16.59)	136	−14.31	(−32.49; 3.88)	210	−6.17	(−22.30; 9.95)
	400–999	104	Ref.		210	Ref.		166	Ref.	
Conflict (ms) ^c^	<400	449	2.44	(−11.16; 16.05)	470	−2.25	(−14.45; 9.96)	440	−5.38	(−17.30; 6.53)
	≥1000	263	2.22	(−11.48; 15.91)	136	0.48	(−13.18; 14.14)	210	−5.78	(−18.74; 7.18)
**N-back task** **(*n* = 673)**										
d’ number 2-back	400–999	86	Ref.		178	Ref.		132	Ref.	
	<400	367	−0.10	(−0.38; 0.17)	378	−0.25 *	(−0.49; 0.01)	358	0.07	(−0.17; 0.31)
	≥1000	220	−0.07	(−0.36; 0.21)	117	−0.13	(−0.40; 0.13)	183	0.12	(0.14; 0.37)
d’ number 3-back	400–999	86	Ref.		178	Ref.		132	Ref.	
	<400	367	0.15	(−0.09; 0.39)	378	0.00	(−0.24; 0.24)	358	−0.01	(−0.21; 0.20)
	≥1000	220	−0.03	(−0.27; 0.22)	117	−0.15	(−0.42; 0.11)	183	−0.19	(−0.41; 0.03)

^a^ Models were adjusted by energy intake (in kilocalories), dietary folate intake per 100 µg/d increase, cohort, social class (I + II (high), III or IV + V (low)), educational level (primary or less, secondary, or university), parity (0 or ≥1), tobacco exposition during the periconceptional period (no or yes), mother’s age (in years), mother’s BMI (continuous), father’s BMI (continuous), and child’s age at 7 y follow-up examination (in years). ^b^ Robust linear regression model was used for hit reaction time and hit reaction time SE (HRT-SE). Negative binomial regression model was used for commission and omission errors (estimates are incidence rate ratios, IRR). Tobit regression model was used for N-back task outcomes and accuracy. ^c^ Higher scores means lower performance. * *p*-value < 0.05. ^#^ I^2^ > 50%, random models were used.

## Data Availability

The data presented in this study are available on request from the corresponding author. The data are not publicly available due to confidentiality and ethical reasons.
